# Individualized Chinese medicine for the treatment of diabetic patients with dry eye disease: A single-case randomized controlled protocol

**DOI:** 10.1097/MD.0000000000018459

**Published:** 2020-01-03

**Authors:** Ruibao Liu, Ying Zhao, Yanxia Wu, Minglu Guo, Yu Duan, Jianbin Ye, Xuejing Lu

**Affiliations:** a Chengdu University of Traditional Chinese Medicine, Academy of Ophthalmology; b Yinhai Eye Hospital affiliated to Chengdu University of Traditional Chinese Medicine, Chengdu, Sichuan, China.

**Keywords:** diabetes mellitus, dry eye disease, individualized treatment, single-case randomized controlled study, traditional Chinese Medicine

## Abstract

**Background:**

The high incidence of Diabetes mellitus (DM) has become a serious challenge for the global epidemic. Increased blood glucose leads to abnormal ocular surface structure and metabolic disorder in patients. DM is a high-risk factor for dry eye disease (DED), with high incidence and increased difficulty in treatment. The disease can cause discomfort, visual impairment, tear film instability and ocular surface damage, and even cause corneal erosion in severe cases, which has a serious impact on people's daily life. Traditional Chinese Medicine (TCM) plays an important role in the evaluation and treatment of DM and its complications. However, whether TCM treatment could improve the treatment efficacy of DM suffering from DED remains poorly understood.

**Objective:**

To investigate the curative effect of TCM for the alleviation of clinical symptoms in Diabetic patients with DED, and to evaluate its long-term efficacy.

**Methods:**

This trial is a single-case randomized, single-blind, placebo-controlled study. A total of 12 subjects will be recruited in this trial. The trial is divided into three cycles, and one cycle has 2 treatment periods. There is a washout period at each adjacent treatment stage. TCM individualized treatment and placebo will be randomized during the treatment period. The test period will last for 29 weeks, with 4 weeks for each treatment period and 1 week for each washout period to minimize carryover effects. Subjects will be selected by the researcher strictly in accordance with the inclusion and exclusion criteria. The outcomes will evaluate the efficacy of treatment by changes in the various observation indicators.

**Discussion:**

This study will realize a patient-centered outcome approach necessary to provide clinical researchers with the evidence that TCM treatment can effectively improve the objective indicators of the eye and systemic symptoms in Diabetic patients with DED.

**Trial registration:**

This study has been registered at the Chinese Clinical Trial Registry (http://www.chictr.org.cn, No. ChiCTR1900024481), (October, 2019).

## 1 Introduction

Diabetes mellitus (DM) has emerged as one of the most major global public health problems,^[1]^ especially in developing nations such as China.^[2]^ It is reported that three in four people living with diabetes (352 million people) are of working age (i.e. between 20 and 64 years old), and this number is expected to increase to 417 million by 2030 and to 486 million by 2045.^[3]^ DM can cause a variety of systemic complications, most of which suffer from dryness of the eye, foreign body sensations, photophobia, and vision fluctuations. During the course of diabetes, microvascular damage to the lacrimal gland due to hyperglycemia, reduced lacrimal innervation as a result of autonomic neuropathy, reduced trophic support to lacrimal tissue, and reduced reflex tearing due to impairment of corneal sensitivity all contribute to the altered tear film status in diabetic patients.^[4]^ The International Dry Eye Workshop 2017 classified diabetes as a risk factor for aqueous-deficient dry eye disease (DED).^[4]^ The incidence of Diabetes combined with DED continues to rise due to changes in lifestyle and dietary preferences in recent years.

DED, also referred to as keratoconjunctivitis sicca, is a complex multifactorial disorder characterized by homeostatic disturbances of the ocular surface and tear film DED is one of the most common eye disorders, and the prevalence of adult populations in different parts of the world is estimated to be between 5% and 50%.^[5]^ There have been reported that the prevalence and severity of dry eye symptoms are higher in Asian ethnic groups than in the Caucasian population.^[6–7]^ The incidence of DED is correlated with the level of glycated hemoglobin: the higher the level of glycated hemoglobin, the higher the incidence of DED.^[8,9]^ Najafi et al. showed that the prevalence of DED in diabetic patients was 27.7%, and DED was significantly associated with glycosylated hemoglobin (HbA1c).^[10]^ Zou et al observed a prevalence of DED was 17.5% in a community-based type 2 diabetic patients, which was lower than that observed in hospital-based studies, but DM patients with poor metabolic control were more likely to develop DED.^[11]^ The course of Diabetes combined with DED is slow, and the inflammatory cascade is triggered by a long-term vicious circle of tear film instability and hyperosmolarity ensues, which may lead to progressive and irreversible changes in the ocular surface.^[12]^ Moreover, DED can have profound effects on the ocular comfort, vision, quality of life, work efficiency of patients, and increase the economic burden for both patients and society. At present, based on the treatment of primary disease, artificial tears are the first choice for the treatment of dry eye among patients with DM, but artificial tears can only relieve the symptoms of patients without treatment from the basic pathological mechanism.^[13]^ Therefore, there is an urgent need to develop novel therapeutic approaches for improving the overall condition of patients with diabetic dry eye.

Laboratory studies and clinical observations have showed that TCM plays an important role in the evaluation and treatment of diabetes and its complications,^[14–16]^ which can assist in delaying or preventing the progression of the disease. In recent years, research has been undertaken to explore the mechanism of action of TCM and its active ingredients, and how to use these active ingredients to develop new treatments for improved management of diabetes and its complications.^[17]^ Qiming Granule (QMG) has the function of Yiqi Shengjin, nourishing liver and kidney, Tongluo Mingmu and it can effectively treat diabetic complications by increasing retinal blood flow and improving the blood circulation to alleviate retinal hypoxia and ischemia.^[14]^ And it has been proved to be suitable for deficiency of qi-yin and blood stasis as the main TCM syndromes in microvascular complications in diabetes.^[14,18,19]^ However, it is unclear whether components in QMG are also effective against Diabetic patients with DED. Therefore, based on QMG, we designed to treat each patient with syndrome differentiation and to form individualized treatment to study its therapeutic effect on Diabetic patients with DED. In our study protocol, a single-case randomized controlled trial (N-of-1) will be used, which can highlight individualized diagnosis and treatment. TCM clinical treatment is based on the principle of “dialectical treatment”, and can determine the treatment plan according to each patient's own situation, which is in line with the features of N-of-1. Therefore, N-of-1 is very suitable for the clinical application of TCM study. Our study aims to evaluate the clinical efficacy of individualized treatment in Diabetic patients with DED and to explore individualized therapeutic assessment methods for TCM research.

## 2 Methods

### 2.1 Trial design

This is a single-case randomized controlled single-blind study. The study population comes from Ineye Hospital affiliated to Chengdu University of Traditional Chinese Medicine. Twelve subjects will be screened for diabetes with DED. The single participant who met the inclusion criteria of TCM clinical response underwent 3 cycles of treatment periods with 6 observation periods, 2 of which are in one cycle, including 4 weeks of TCM individualized treatment and 4 weeks of placebo. In each cycle of testing, each subject determines the order in which the two drugs are used simulant at random. All participants will continue their basic treatment during entire the trial period. There is no washout period at the beginning of the study and there is 1 week washout period at each adjacent treatment stage to make carryover effects minimized and observe the disease development.

**Figure 1:**
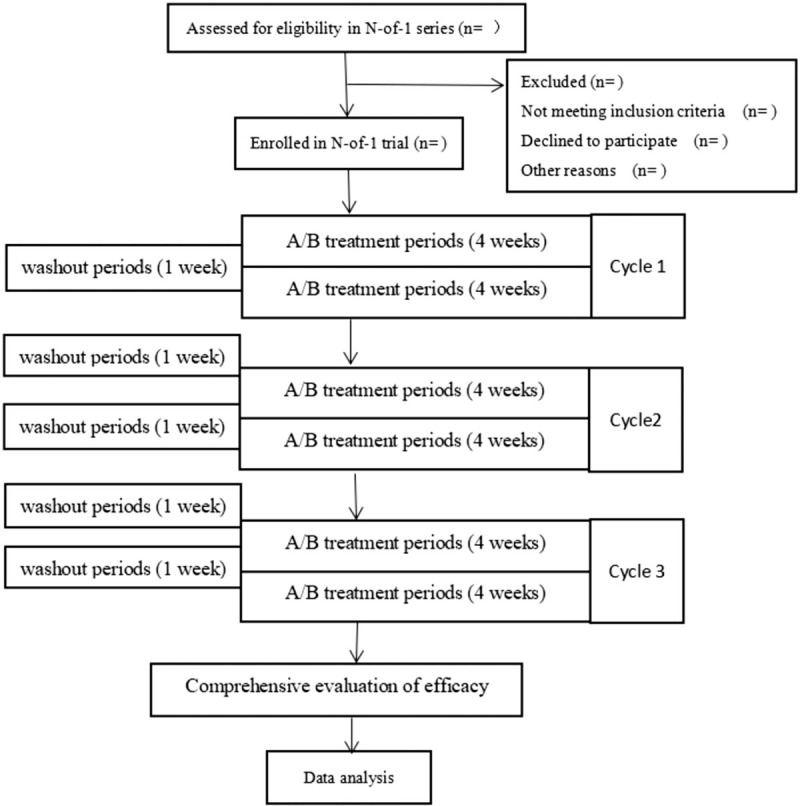
Flow Chart of Clinical Trial in Diabetes with dry eye disease. Note. The trial consisted of 3 cycles and is divided into six treatment periods. A/B treatment represents patients receiving TCM individualized treatment and placebo treatment are randomized. There are 4 weeks for each treatment period and a 1-week washout period at the end of treatment.

The study will be conducted in October and November, 2019. The intervention will begin in November and December 2019 and it is planned to be concluded by September and October, 2019. The entire study experiment time is 29 weeks. Figure 1 shows the overall flow of the subject's drug treatment process. The protocol is compliant with the Standard Protocol Items: CONSORT extension for reporting N-of-1 trials (CENT) 2015.^[20]^ If the symptoms change and develop, patients will be offered the option of stopping a treatment period early and waiting for the other treatment period after a washout period or withdrawing the trial.

Due to the complex complexity of herbal ingredients, their onset time and half-life are not easily predicted from their pharmacokinetics or pharmacodynamics and a possible residual effect might be expected. Therefore, the treatment periods of each two adjacent pair will be separated by 1-week wash-out interval to minimize carryover effects.

### 2.2 Classification of dry eye syndrome

DED can be divided into 2 types: aqueous tear-deficient (tear-deficient, lacrimal tear deficiency) and evaporative dry eye. Aqueous-deficient dry eye has two major subgroups: Sjögren and non-Sjögren syndrome. Evaporative dry eye may be intrinsic (e.g., due to meibomian gland dysfunction, low blink rate, or eyelid problems) or extrinsic (e.g., due to contact lens wear, preservatives in topical medications, or diseases of ocular surgeries).^[21]^ DM associated dry eye may be tear-deficient or evaporative dry eye.^[22]^

### 2.3 Study population

#### 2.3.1 Diagnostic criteria

The definition criteria of DED is based on the revised Japanese DED diagnostic criteria:

(1) the symptoms of dry eye diseases;
(2) presence of either a quantitative or qualitative disturbance of the tear film (BUT ≤5 s or Schirmer I test ≤5 mm); and
(3) presence of conjunctivo-corneal epithelial damage (fluorescein staining score ≥ 3 points, Rose Bengal staining score ≥ 3points, or lissamine green staining score ≥ 3 points).

A definitive diagnosis of DED was established only if all three criteria were present.^[23]^ New criteria for diagnosis and treatment of DM will be referred to the American Diabetes Association (ADA) criteria.^[24]^

The TCM syndrome differentiation type of deficiency of both qi-yin and blood stasis are based on the guidelines of TCM.^[25]^ The primary signs and symptoms include conscious dry eyes, a gritty or foreign body sensation, fatigue, burning sensation, eye irritation, tearing, photophobia, visual fluctuations, stinging or intermittent sharp pain (The first 3 items are required). The secondary signs and symptoms include

(a) qi-deficiency: shortness of breath, low voice or disinclination to talk, tired, lack of strength, spontaneous sweating;
(b) yin-deficiency: thirst with liking for fluids, vexing heat in chest, palms and soles, dizziness and tinnitus, night sweats, constipation;
(c) pale or dark tongue or petechia, little coating, thready and uneven pulse.

Among the above symptoms, Participants who suffer with the main primary signs and 3 to 4 species in each secondary symptom, combined with the tongue and pulse can be diagnosed with deficiency of both qi-yin and blood stasis.

#### 2.3.2 Inclusion criteria

Patients who meet the following criteria will be considered for enrollment:

(a) patients with confirmed diabetes combined with DED;
(b) comply with TCM syndrome differentiation standards;
(c) between 18 and 75 years of age;
(d) accepted the survey voluntarily and signed informed consent;
(e) ability to return for study visits.

#### 2.3.3 Exclusion criteria

Patients were excluded from participation in the study if any of the exclusion criteria were met:

(a) women who were pregnant or breastfeeding;
(b) patients with any ocular surface disease, scarring, acute ocular infection, blepharitis, and an unwillingness to discontinue contact lens wear during the study;
(c) receive any eye surgery within 3 months;
(d) history of diseases that affect vital organs, including heart, liver, and kidney;
(e) mental disorder;
(f) participated in other clinical trials in recent 3 months.

#### 2.3.4 Withdrawal criteria

Participants who meet any of the following conditions will be removed from the study:

(a) Patients with poor compliance who did not take drugs according to requirements of the study protocol were rejected;
(b) voluntary withdrawal from the trial;
(c) intolerant or allergic to the drug.

#### 2.3.5 Participant recruitment

The trial will be set up and co-ordinated by academy of ophthalmology of Chengdu University of TCM. Subjects will be recruited from Ineye Hospital affiliated to Chengdu University of TCM, or they can be recruited online or by advertising.

### 2.4 Intervention

All subjects in the trial will continue to receive basic medicine. After completing the TCM syndrome differentiation, the doctor gives the individualized treatment prescription and the control prescription (placebo). The individualized treatment will follow routine practice of Chinese herbal medicine (CHM). Both the drugs are Chinese medicine granules and uniformly processed and provided by the pharmacy of Ineye Hospital affiliated to Chengdu University of TCM. Written consent will be obtained from all participants prior to the start of the program.

Experimental group: QMG consists of 8 herbs, namely Astragalus membranaceus, Radix Rehmanniae, Fructus Lycii, Radix Puerariae, Cassia Seed, Cattail Pollen, Motherwort Fruit and Hirudo. The individualized TCM are as follows: blood deficiency, plus white peony, Angelica; yin deficiency and fire wang signs clearly, plus Anemarrhena asphodeloides, Cortex Phellodendri; qi deficiency, plus Radix ginseng, Schisandra; blood stasis, plus Panax notoginseng, Salvia Miltiorrhiza; yang deficiency, plus Eucommia, Herba Epimedii; turbid-phlegm, plus Rhizoma Pinelliae, Poria cocos; water-dampness, plus Grifola umbellata, Alisma; qi stagnation, plus Fructus aurantii, Dried tangerine peel. The therapeutic formula will be adjusted based on the patient's clinical performance each treatment cycle.

Placebo control group: The preparation method of traditional Chinese medicine placebo is made by using a low proportion of drugs and auxiliary materials, and 10% of the test drugs are used to increase the similarity. Lactose is mixed with flour as a diluent, edible caramel, sunset yellow and lemon yellow pigment are used as a coloring agent, and edible bittering agent and aspartame are used as a flavoring agent. Made by mixing, drying, pulverizing, sieving, granulating, and finishing. It is basically consistent with the test drugs in terms of appearance, smell, color, taste and packaging. Both groups of drugs are provided by Sichuan New Green Pharmaceutical Technology Development Co., Ltd. Method of administration: took mixed with warm boiled water, 3 times a day.

### 2.5 Outcomes

Patients need to be at baseline, treatment week 4, week 5, week 9, week 10, week 14, week 15, week 19, week 20, week 24, week 25, and week 29 visits. Various eye examinations are performed on the right eye of each patient and each visit requires observation and recording of measurement indicators. An overview of specific measurements and time points of data collection are provided in Table 1.

**Table 1:**
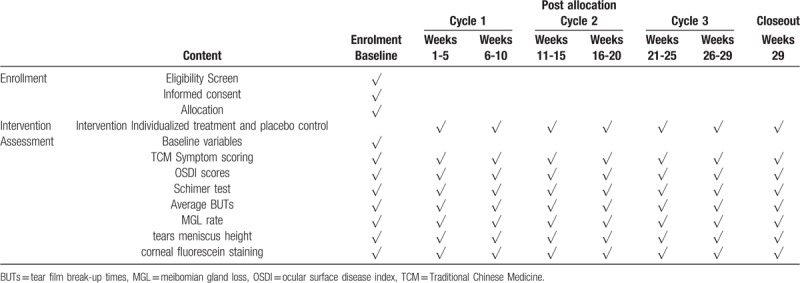
Time points and content of on-site data collection.

#### 2.5.1 Symptom scoring

Patients were required to fill out a questionnaire, including TCM syndrome scores and symptom survey with the 12 Ocular Surface Disease Index (OSDI) items.^[26]^

#### 2.5.2 Eye examination

Schirmer I tests (without anesthesia): performed by placing a strip in the unanesthetized eye over the lower lid margin, midway between the middle and outer third and recording the length of the strip that was wetted after 5 minutes.

The noninvasive tear film break-up time (NITBUT): NITBUT measurements are evaluated using the Keratograph 5 M (OCULUS Optikgeräte GmbH, Wetzlar, Germany). The professional operator will note the first NITBUT (NITBUTf), and the average value of the intervals after blinking on the corneal viewing area are recorded as the average NITBUT (NITBUTav).

Meibomian gland loss: Images showing Meibomian gland loss of the right upper eyelid were obtained using the OCULUS Keratograph ocular surface analyzer in accordance with the manufacturer's instructions. Analyze and record the ratio of meibomian gland dropout area to the total area.

Fluorescein staining: Corneal-conjunctiva staining was accessed using the cobalt-blue filter of the slit-lamp light source through a Wratten 12 yellow filter, 2 minutes after instilling 5 mL of 2% sodium fluorescein. The eye is divided into three equal compartments: the cornea, the nasal conjunctiva and the temporal conjunctiva. The staining of each region is graded from 0 to 3 points. A score of 0 represented no staining; a score of 1 represented 1-30 staining dots; a score of 2 scores represented > 30 staining dots; a score of 3 scores represented total staining with ulcer and filaments. The final stain scores are the sum of the ranks of the three regions.

#### 2.5.3 Safety indicator

Laboratory tests including blood routine and urine routine, liver and kidney function, blood lipids, 12-lead ECG, electrocardiogram, etc. and blood pressure, respiration, heart rate, and body temperature are measured before and after the study. Any adverse events or adverse reactions related to the treatment method or drugs are observed, and the study will be terminated if necessary.

#### 2.5.4 Safety assessment

During the entire study period, any adverse events will be documented and reported in detail. Protocols have also been designed to support researchers in situations where participants becomes distressed or presents with risks to self or others.

### 2.6 Sample size

In an N-1 design that is repeated between participants, the size of the sample (series) is not based on the ability to test group effects using inferential statistics. And the examinations for each individual trial may change over time, allowing us to use predefined criteria to determine if treatment is effective for each subject. In this study, we selected 12 samples (N trials, 12 replicates) with at least four measurement points per cycle. This sample size will be sufficient to provide information on whether the intervention is applicable to the individual to examine the direction of the “average therapeutic effect” between individuals and to provide an estimate of the variability of intra- and inter-individual outcomes in that population. As a standard for the N-of-1 trial, this number of participants will allow for statistical analysis of the magnitude of the effect of the intervention relative to the baseline.

### 2.7 Randomization and blinding

A total of 40 sets of random numbers are generated by www.randomizer.org. Every odd number is designated as the individualized treatment prescription and even number is designated as the control prescription (placebo). The random orders of the two treatment formulations are allocated by opaque envelope. We estimate to recruit 12 participants in the feasibility study and use 36 sets. Other 4 sets are spare. The test drugs were randomly assigned by the database administrator according to the coding table. The prepared research drugs will be delivered, stored, and distributed in the pharmacy of Ineye Hospital affiliated to Chengdu University of TCM. Both the drugs have no differences in appearance, dosage form, color, specification, and so forth. Due to the special nature of TCM treatment, double-blind cannot be achieved. The patients, data managers and outcome assessors will be blinded during the trial.

### 2.8 Adherence to study medication

Study drug compliance will be assessed by:

(1) weekly self-recorded drug intakes;
(2) the trial staff will ask questions about compliance during the planned telephone consultation from the first week after the start of the review and
(3) The number of drug bags returned after the treatment is completed. At the end of the treatment period, participants will be asked to return all unused medication for counting in a reply-paid post satchel.

### 2.9 Data collection and management

The trial will set up the project Executive Committee (EC), Monitor Group (MG) and Data and Safety Monitoring Group (DSMG). All the data is obtained from the research medical record, which is flled in by the researcher. The data is collected through the electronic case report form (e-CRF) that must be completed for each selected case. Create a dedicated database for data entry and management and the data manager is responsible for data management. Data entry is done by trained personnel and performs data quality and accuracy checks. Two data administrators should perform 2 separate inputs and proofreading to ensure the accuracy of the data.

### 2.10 Quality assurance and data integrity

The trial will be conducted in accordance with the protocol, the ICH Good Clinical Practice Guide and all relevant local ethical regulations. Data collection will be performed in accordance with the pre-approved protocols, and the quality of the study will be managed by regular monitoring-MG. The integrity of the test data will be monitored by periodically checking for omissions and errors in the data sheet.

### 2.11 Statistical analysis

All data analyses are performed using SAS. A population estimate of effect by aggregating individual N-of-1 results was used. The results of all complete cycles were combined to produce a posterior probability of the overall difference between TCM individualized treatment cycles and placebo cycles. For all analyses, *P* *<* .05 is considered statistically significant.

## 3 Discussion

DM is a clinically common chronic disease, like high blood pressure and coronary heart disease, which can cause a series of complications such as neovascular glaucoma, cataract, etc.^[27]^ In recent years, patients with DM have known to have an increased prevalence of ocular surface disease and DED.^[28]^ DM is one of the most frequent causes of DED, which corresponds to the increased incidence and severity of long-term DED.^[29]^ In DM patients, tear film instability and hyperosmolarity can be due to higher levels of glucose and protein in tear fluid and alterations in the protein profile.^[30]^ Suzuki et al^[31]^ used the improved DEWS scale to evaluate the association between tear film osmolarity (TFO) and DED severity and found that TFO was significantly associated with DED severity. DM patients with good glycemic regulation can reduce their TFO and OSDI scores to improve tear function.^[32]^ The future of diabetes management depends on an increased awareness of the importance of diabetic ocular surface, and both DM and DED seriously affect the quality of life of patients. Therefore, there is an urgent need to develop novel therapeutic approaches for the treatment of diabetes associated with DED.

This is a randomized-controlled, double-blind, single-case clinical trial in which each subject can be considered as having its own RCT. Compared with traditional RCT, N of 1 is an overall assessment of the effectiveness of current medical measures which maximizes reference to the subject's opinion and is truly “patient-centered”. The research process is completely in line with the clinical diagnosis and treatment model of TCM,^[33]^ but N of 1 trial use self-control and may lead to false negative results. Therefore, the entire process needs to be strictly carried out.

TCM theory holds that “Qi is the essence of the body”, which means that blood flow and fluid exchange of blood are dependent on the promotion of Qi. Qi stagnation or qi deficiency can make it difficult to promote blood circulation, which in turn increases the problem of Qi. This study aimed at the common pathogenesis of “deficiency of qi-yin and blood stasis”, and established the idea of treating diabetes associated with DED based on Chinese herbal medicine of Yiqi Shengjin, nourishing liver and kidney, Tongluo Mingmu. The efficacy of TCM in the treatment of Diabetes with DED was evaluated by observing various objective indicators. At the same time, we believe that dry eye related examination should be used as a routine examination of DM patients, in order to facilitate the early detection of positive indicators to improve the patient's self-eye comfort and quality of life.

## 4 Ethical approval and trial registration

The protocol has been approved by the Ethics Committee of Ethics Review Committee of Ineye Hospital of Chengdu University of TCM (No. 2019yh-005) and registered with the Chinese Clinical Trial Registry (http://www.chictr.org.cn, No. ChiCTR1900024481). All patients will be invited to provide a signed informed consent to participate in the study.

## Author contributions

**Analysis**: Yanxia Wu, Yu Duan.

**Conceptualization**: Ruibao Liu, Ying Zhao.

**Formal analysis**: Ruibao Liu, Xuejing Lu.

**Funding acquisition**: Xuejing Lu.

**Investigation**: Yu Duan. Minglu Guo, Yanxia Wu, Ying Zhao, Jianbin Ye.

**Methodology**: Ruibao Liu, Xuejing Lu.

**Project administration**: Xuejing Lu, Ruibao Liu.

**Supervision**: Ying Zhao, Xuejing Lu.

**Writing – original draft**: Ruibao Liu.

**Writing – review & editing**: Ying Zhao, Yanxia Wu, Minglu Guo, Yu Duan, Jianbin Ye, Xuejing Lu.
